# A rare case report: Gallbladder-associated ectopic liver tissue: Challenges, insights, and surgical considerations

**DOI:** 10.1016/j.ijscr.2024.109261

**Published:** 2024-01-12

**Authors:** Wael Ferjaoui, Ahmed Omry, Amel Changuel, Khouloud Mejri, Med Hedi Mannai, Med Bachir Khalifa

**Affiliations:** a General Surgery Department, Military Hospital of Tunis, Mont Fleury-1008, Tunis, Tunisia; b Faculty of Medicine of Tunis, 15, Djebel Lakhdhar Street – 1007 Bab Saadoun, Tunis, Tunisia

**Keywords:** Case report, Ectopic liver tissue, Gallbladder, Hepatocellular carcinoma, Laparoscopy cholecystectomy

## Abstract

**Introduction and importance:**

Ectopic liver tissue (ELT), a rare anomaly distinct from accessory liver, challenges conventional embryonic morphogenesis. Unlike the latter, ELT lacks a connection to the main liver, showcasing an unusual growth of normal liver tissue beyond its customary location. This peculiarity poses clinical and radiological challenges for surgeons throughout their careers, particularly during laparoscopic or open procedures. Elevated clinical significance arises from ELT's potential to progress into hepatocellular carcinoma, necessitating heightened awareness among surgeons.

**Case report:**

This article presents a compelling case of ELT, discovered incidentally during a planned laparoscopic cholecystectomy. The patient, a 60-year-old female with a history of biliary colic, underwent a meticulous exploration revealing an undistended gallbladder with an unexpected brownish tissue fragment resembling hepatic parenchyma.

**Clinical discussion:**

Ectopic liver tissue, dating back to early 20th-century records, challenges precise incidence determination. Theories regarding embryonic development around the fourth week in utero provide insights into ELT's origins and displacement from the hepatic diverticulum. Varied attachment locations and potential manifestations in other intra-abdominal and intra-thoracic sites add layers to the complexity of its diagnosis. Radiological studies, though challenging, offer glimpses of ELT, cautioning against percutaneous biopsies due to associated risks.

**Conclusion:**

In conclusion, this case of ELT offers valuable insights into its diagnostic challenges and surgical considerations, underscoring the need for continued research and heightened awareness in the medical community. The rarity and varied presentations of ELT warrant ongoing exploration to refine diagnostic approaches and optimize patient outcomes.

## Highlights

• Emphasizing the rarity of gallbladder-associated ectopic liver tissue (ELT) within cholecystectomy series, our case stands as a singular occurrence in a sizable dataset.
• Navigating the surgical landscape, we highlight the unique considerations and potential complications posed by ELT during laparoscopic cholecystectomy, urging surgeons to exercise heightened vigilance.
• Bringing attention to the diagnostic challenges surrounding ELT, we illuminate the potential oversight in radiological studies, stressing the imperative for increased awareness among healthcare professionals.
• Beyond its diagnostic intricacies, our case underscores the clinical implications of ELT, not only in its varied intra-abdominal locations but also in its association with congenital anomalies, urging a comprehensive understanding for optimal patient management.

## 1 Introduction

Ectopic liver tissue (ELT) is a rare condition, differing from accessory liver in that it is not linked to the main liver [1,2]. It results from a morphogenesis error, where the growth of regular liver tissue takes place outside its usual anatomical location [3,4]. ELT may pose a clinical or radiological challenge for surgeons at any point in their career and can be encountered during laparoscopic or open surgical procedures [5]. Ectopic liver tissue has been identified both above and below the diaphragm, with the gallbladder-associated ectopic liver being the most prevalent intra-abdominal location [1,5]. Furthermore, given its tendency to lead to hepatocellular carcinoma, the recognition of ELT should be of clinical significance, emphasizing the importance of surgeons being aware of this potential variation [3,6]. To underscore its importance, this paper presents a case of ectopic liver attached to the gallbladder serosa, which was incidentally discovered during a planned laparoscopic cholecystectomy.

This work has been reported in line with the SCARE 2023 criteria [7].

## 2 Case presentation

**Fig. 1:**
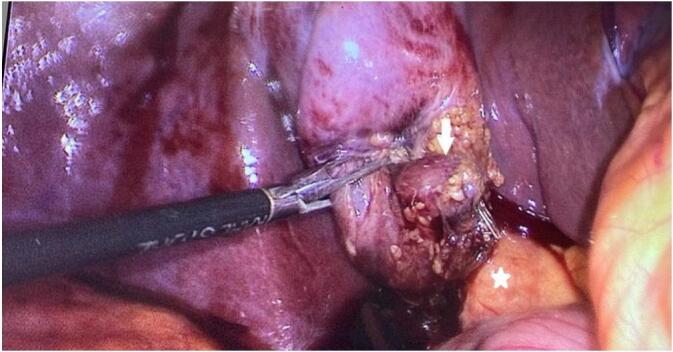
Laparoscopic view of ectopic liver attached to the anterior surface of the gallbladder wall neck (white arrow) near the Mascagni's lymph node (white star).

**Fig. 2:**
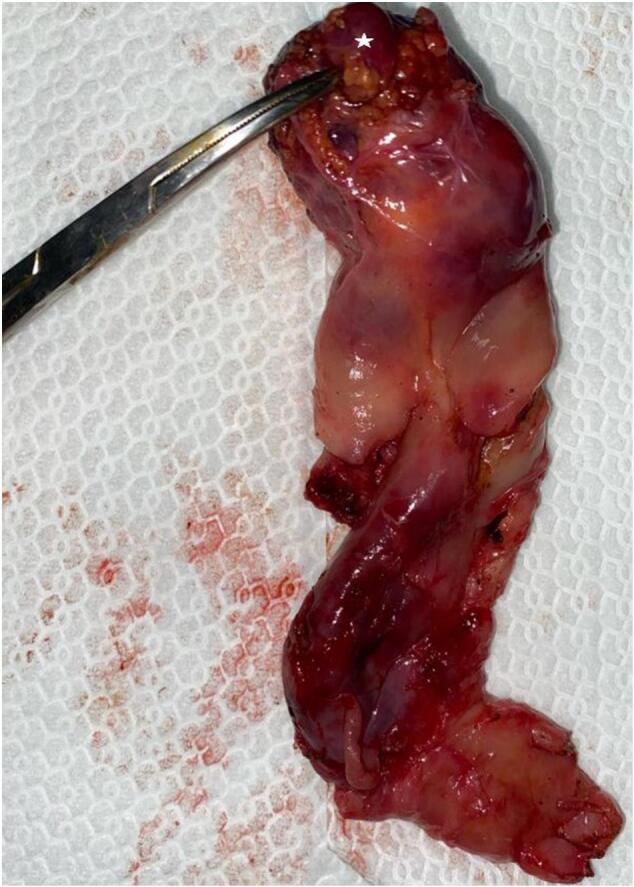
Gallbladder removed by laparoscopic approach containing ectopic liver measuring 20 mm × 10 mm × 5 mm firmly attached to the peritonized surface of the gallbladder wall (white star).

A 60-year-old female with no prior medical or surgical history, presented 10-month history of biliary colic exacerbated with fatty meals and relieved with medications. The interview did not identify recent jaundice or other associated functional signs. The physical examination revealed normal-colored conjunctivae. Her abdomen was soft and depressible without palpable mass. The laboratory tests were normal, including normal liver function tests. The preoperative abdominal ultrasound showed an undistended gallbladder with a thin wall containing a large calculus of 2 cm. The liver appeared normal in morphology and size. The patient underwent elective laparoscopic cholecystectomy. The intraoperative exploration revealed an undistended gallbladder with edematous wall. A brownish tissue fragment resembling hepatic parenchyma was identified on the anterior wall of the gallbladder neck near the Mascagni's lymph node. Its identification was based on its distinct color. It measured 2*1 cm in size (Fig. 1). It possessed an independent vascular pedicle originating from the cystic artery, but there was no connection for biliary drainage to the main biliary system or cystic duct. The vascular pedicle was separately isolated and clipped. A retrograde cholecystectomy was performed, removing this tissue fragment. Upon opening, the gallbladder revealed a brown-yellowish gallstone measuring 20 mm in diameter, adhered to the luminal surface. The excised specimen was forwarded to the histopathology department (Fig. 2). Microscopic analysis verified the existence of chronic inflammation with ectopic liver tissue adhering to the gallbladder serosa. Sinusoidal congestion, mild steatosis, and focal deposits of hemosiderin were observed, with no evidence of malignant degeneration. The postoperative course was uneventful, and the patient was discharged on the first postoperative day.

## 3 Discussion

Ectopic liver tissue is an exceptionally uncommon occurrence, with the earliest recorded data dating back to the 1920s [1]. Determining the actual incidence of ectopic liver tissue attached to the gallbladder wall is challenging, as the majority of cases are asymptomatic and are typically identified during laparotomy, laparoscopy, or autopsy [3,5]. While the precise incidence of ectopic liver tissue (ELT) remains uncertain, two extensive studies on this subject revealed three cases (0.05 %) during autopsy among 5500 cases and twelve cases (0.6 %) during laparoscopy among 1802 cases associated with gallbladder [1,3]. In this current report, our patient constituted the sole case (0.1 %) of ectopic liver tissue among 911 cholecystectomy cases over a two-year period. Various theories exist to elucidate the occurrence of ectopic liver [8]. Nevertheless, it is predominantly postulated to originate around the fourth week in utero during the embryonic development of the liver [1,8]. This development occurs due to the displacement of a segment of the cranial part of the hepatic diverticulum from the liver bud to alternative locations [8]. The proximity of the developing hepatic parenchyma cell cords to the pars cystica may provide an explanation for the presence of ectopic hepatic tissue in the gallbladder wall [3]. While ectopic liver tissue typically attaches to the gallbladder's serosa or resides within its wall, it can also manifest within the gallbladder lumen [8]. Ectopic liver tissue is occasionally linked to additional congenital anomalies, including biliary atresia, agenesis of the caudate lobe, omphalocele, bile duct cysts, or cardiac anomalies [3]. Despite the most common localization of ectopic liver tissue (ELT) in the gallbladder, it can be observed in both the intra-abdominal and intra-thoracic cavities [1]. Instances have been identified in the spleen, umbilicus, vena cava, as well as the heart and lungs [1,2]. Detecting ectopic liver tissue (ELT) without resorting to surgery or autopsy poses a challenge through radiological studies [3]. The rarity of this condition and the lack of awareness may contribute to radiologists potentially overlooking it [3,5]. It may manifest as a soft tissue mass in CT or US imaging [3]. Performing a percutaneous biopsy is not advised due to the associated risks of malignancy and bleeding [1,3]. In the patient described in the present report, the ultrasonographic examination before surgery did not show signs in favor of EL. Ectopic liver tissue attached to the gallbladder typically remains asymptomatic and is occasionally identified during laparoscopy, as illustrated in the current case [5,8]. When symptoms do occur, the primary complaint is typically upper abdominal pain, arising from complications such as torsion, hemorrhagic necrosis, rupture, or compression by the mass, often associated with malignant transformation to hepatocellular carcinoma (HCC) [1,8]. The histological characteristics of ectopic liver (EL) tissue closely resemble those of the normal liver, exhibiting regular lobules, a central vein, and normal portal spaces [2,3]. In certain instances, an elevated number of blood vessels on the outer surface of the gallbladder attached to the ectopic liver tissue can be observed, akin to the findings in our patient [3]. It is important to identify ectopic liver tissue (ELT) and its vascular supply prior to dissecting the gallbladder from the liver bed [1]. Failure to do so may lead to inadvertent traction on the gallbladder during removal, potentially causing rupture or tearing of vascular structures directly derived from the liver substance and, consequently, resulting in severe bleeding [1]. The vascular peduncle typically arises either directly from the liver substance or from the cystic artery [1]. In the context of the presented case, emphasizing the importance of meticulous dissection and respect for the critical view of safety during cholecystectomy becomes paramount [1,2]. Following the dissection of Calot's triangle, it is strongly recommended to meticulously isolate and clip the vascular peduncles or mesentery individually before proceeding with gallbladder dissection, as exemplified in our approach [1,6]. This strategy aligns with established safety practices and minimizes the risk of inadvertent injury, advocating for a methodological adherence to cholecystectomy with a critical view of safety over the classic retrograde cholecystectomy approach under such circumstances [[1], [2], [3]].

## 4 Conclusion

In conclusion, ectopic liver tissue (ELT) remains a rare and clinically significant entity, often presenting a diagnostic challenge [1,2,4]. Our case, featuring ELT attached to the gallbladder serosa, emphasizes the importance of awareness among surgeons during laparoscopic procedures [3]. The prevalence of ELT, though uncertain, underscores its rarity, with our patient constituting a singular case in a sizable cholecystectomy series. The histopathological analysis revealed typical features of ELT, highlighting its resemblance to normal liver tissue [1,3,4]. Recognition of ELT's potential association with congenital anomalies and its varied intra-abdominal and intra-thoracic locations is crucial for comprehensive patient management [8]. Surgical precautions, including vascular peduncle identification, are essential to prevent complications such as bleeding [1].

## Patient consent

Written informed consent was obtained from the patient for the publication of this case report and its accompanying images. A copy of the written consent is available for the Editor-in-Chief of this journal to review upon request.

## Ethical approval

Ethical approval is not applicable/waived at our institution. Due to the specific nature of case reports, which involve detailed descriptions of observations and interventions that have already been conducted on patients, as opposed to prospective studies involving planned interventions, our institution does not require formal ethical approval for such cases. We recognize the importance of ethics in medical research and are fully committed to upholding ethical standards in our medical and research practices.

## Funding

This research did not receive funding from any specific grant provided by public, commercial, or not-for-profit organizations.

## Author contribution

Wael Ferjaoui and Ahmed Omry contributed to manuscript writing and editing, and data collection; Khouloud Mejri and Amel Changuel contributed to data analysis; Med Hedi Mannai and Med Bachir Khalifa contributed to conceptualization and supervision; All authors have read and approved the final manuscript.

## Guarantor

Dr. Ahmed Omry.

## Research registration number

NA.

## Declaration of generative AI in scientific writing

AI tools were not used for the elaboration of the manuscript.

## Conflict of interest statement

No conflicts of interest.
